# Gaucher disease and the synucleinopathies: refining the relationship

**DOI:** 10.1186/1750-1172-7-12

**Published:** 2012-01-31

**Authors:** Tessa N Campbell, Francis YM Choy

**Affiliations:** 1 TNC Scientific Consulting, Calgary, AB, Canada; 2 Department of Biology, University of Victoria, PO Box 3020, Station CSC, Victoria, BC, V8W 3N5, Canada

**Keywords:** Gaucher disease, glucocerebrosidase, *GBA *mutations, lysosomal storage disease, synucleinopathies, Parkinson's disease, dementia with Lewy bodies, multiple system atrophy, neurodegeneration with brain iron accumulation, protein misfolding

## Abstract

Gaucher disease (OMIM 230800, 230900, 231000), the most common lysosomal storage disorder, is due to a deficiency in the enzyme glucocerebrosidase. Gaucher patients display a wide spectrum of clinical presentation, with hepatosplenomegaly, haematological changes, and orthopaedic complications being the predominant symptoms. Gaucher disease is classified into three broad phenotypes based upon the presence or absence of neurological involvement: Type 1 (non-neuronopathic), Type 2 (acute neuronopathic), and Type 3 (subacute neuronopathic). Nearly 300 mutations have been identified in Gaucher patients, with the majority being missense mutations. Though studies of genotype-to-phenotype correlations have revealed significant heterogeneity, some consistent patterns have emerged to inform prognostic and therapeutic decisions. Recent research has highlighted a potential role for Gaucher disease in other comorbidities such as cancer and Parkinson's Disease. In this review, we will examine the potential relationship between Gaucher disease and the synucleinopathies, a group of neurodegenerative disorders characterized by the development of intracellular aggregates of α-synuclein. Possible mechanisms of interaction will be discussed.

## Review

### Overview of Gaucher Disease

Gaucher disease (OMIM 230800, 230900, 231000), the most common lysosomal storage disorder, is characterized by a spectrum of signs and symptoms caused by the defective hydrolysis of glucocerebroside. A deficiency in the enzyme glucocerebrosidase (GBA, glucosylceramidase, acid β-glucosidase, EC.3.2.1.45) leads to the accumulation of its glucocerebroside substrate in the liver, spleen, and bone marrow. The predominant symptoms are hepatosplenomegaly, haematological changes, and orthopaedic complications [1,2]. Gaucher disease has been classified into three phenotypes based upon the presence or absence of neurological involvement: Type 1 (non-neuronopathic; most common form), Type 2 (acute neuronopathic) and Type 3 (subacute neuronopathic) [3].

The *GBA *gene, located on chromosome 1q21-22, is comprised of 11 exons encoding a 497 amino acid protein. Presently, nearly 300 mutations have been identified in Gaucher patients, including frame-shift mutations, point mutations, deletions, insertions, splice site mutations, and recombinant alleles [2,4,5]. For the purpose of genotype-phenotype correlations, many of these mutations have been classified as "null," "severe," or "mild" with respect to levels of glucocerebrosidase production. Null mutations, such as c.84dupG (84 GG), do not direct any enzyme production. Severe mutations, such as c.1448T > C (L444P), produce enzyme but, when inherited with a null or another severe mutation, are usually associated with Type 2 or 3 disease. Mild mutations, such as c.1226A > G (N370S), are those that are only associated with Type 1 disease [6].

Gaucher disease is the first lysosomal storage disorder to be successfully treated by enzyme replacement therapy [7]. At present, alglucerase (Ceredase^®^, Genzyme Inc.), imiglucerase (Cerezyme^®^, Genzyme Inc.), and velaglucerase alfa (VPRIV™, Shire) have been FDA-approved for treatment of Gaucher patients [8,9]. Alternative therapies have also been developed. In 2003, substrate reduction/inhibition therapy (miglustat, Zavesca^®^, Actelion Pharmaceuticals) was FDA-approved for adult patients unsuitable for enzyme replacement therapy [10]. Other treatment avenues under exploration are stabilization of the mutant lysosomal protein through chaperone therapy and introduction of wildtype glucocerebrosidase genes through gene therapy [11].

Recent research has highlighted a potential role for Gaucher disease in other comorbidities such as cancer and Parkinson's disease. In this review, we discuss the emerging relationship between Gaucher disease and the synucleinopathies, a group of neurodegenerative disorders characterized by the development of intracellular aggregates of α-synuclein.

### Overview of the Synucleinopathies

The synucleinopathies encompass a group of various neurodegenerative disorders that share a common pathologic lesion comprised of aggregates of α-synuclein protein in vulnerable populations of neurons and glia [12]. The synucleinopathies discussed in this review are Parkinson's disease, dementia with Lewy bodies, multiple system atrophy, and neurodegeneration with brain iron accumulation.

The synuclein family consists of soluble proteins characterized by an acidic carboxyl terminus and five to six imperfect repeat motifs (KTKEGV) distributed throughout the amino-terminus. The members range in length from 127 to 140 amino acids [12]. Initially described in 1988, the first synuclein family member (α-synuclein) was purified from the Torpedo electroplaque and from rat brain [13,14]. It was also later named the nonamyloid component (NAC) of plaque precursor protein after the NAC peptide was isolated from amyloid-rich senile plaques of Alzheimer patient brains [12,14]. The α-synuclein gene has been mapped to chromosome 4q21.3-q22 [14,15]. There are currently three additional members of the synuclein family: β-synuclein, γ-synuclein, and synoretin. The functions of the synuclein family members remain poorly understood [12].

### Gaucher Disease and Parkinson's Disease

Parkinson's disease (PD) is the second most common neurodegenerative disorder, with greater than 1% affected over 65 years of age and more than 4% of the population affected by the age of 85 years [16,17]. Research indicates that PD likely results from a combination of polygenic inheritance, environmental exposure, and gene-environment interactions. Approximately 20% of PD patients report a family history of the disease [17,18]. Traditionally, PD has been defined by the presence of classic motor signs: rigidity, tremor, bradykinesia, and postural instability. However, recent evidence indicates that nonmotor characteristics such as autonomic insufficiency, cognitive impairment, olfactory deficits, psychosis, depression, and sleep disturbance are also common occurrences [17]. The first gene (*SNCA*, PARK1 locus) causally linked to PD was discovered via analysis of a large multigenerational Italian family in which parkinsonism segregated in an autosomal dominant pattern [19,20]. Subsequently, a total of 18 PD loci (PARK 1-18) have been proposed through linkage analysis and genome-wide association studies [17]. Mutations within genes at six of these loci (*SNCA, LRRK2, PRKN, DJ1, PINK1*, and *ATP13A2*) have been directly linked to familial parkinsonism [21]. Recently, specific variations in the Gaucher disease-associated gene *GBA*, which is not assigned to a PARK locus, have been suggested as risk factors for PD, as discussed below [22].

Over the past decade, several lines of evidence have emerged implicating an association between parkinsonism and mutations in the glucocerebrosidase gene. Recognition of the relationship between *GBA *mutations and PD initially began in the clinic, with the identification of rare Gaucher patients with parkinsonian symptoms appearing in case reports, larger patient series, and prospective studies [22]. Moreover, pedigree analyses indicated an elevated incidence of Parkinson's disease in relatives of Gaucher patients, many of whom were obligate heterozygotes [23,24]. Additionally, multiple independent studies surfaced reporting an increased frequency of *GBA *mutations in different cohorts with parkinsonism [25-30]. Despite this evidence, early studies were often constrained by small sample sizes or evaluation of only a few common *GBA *mutations [31], complicating a consensus to label *GBA *mutations as risk factors for typical Parkinson's disease. In 2009, Sidransky et al. [22] published a hallmark study on this topic: a collective analysis of 5691 patients with PD complemented by 4898 controls from 16 centers across 12 countries. For the pool of participants in which the full *GBA *coding region was screened, loss-of-function mutations were observed in 6.9% of cases and 1.3% of controls (odds ratio, 5.4; 95% CI, 3.9-7.6). Among the Ashkenazi Jewish subset, higher mutation frequencies were seen: 19.3% in cases and 4.1% in controls [17,22]. The findings were not exclusive to a specific ethnicity, nor associated with any particular *GBA *mutation. Additional noted trends were: subjects carrying mutations presented an average of four years earlier, were more likely to have a family history of PD, and had less bradykinesia and rest tremor and more cognitive changes described [22]. Other cohort studies have corroborated the results from this collaborative examination, reinforcing mutations in *GBA *as the number one genetic risk factor for PD [22,32-34].

### Gaucher Disease and Dementia with Lewy Bodies

Like Parkinson's disease, dementia with Lewy bodies (DLB) is a common neurodegenerative condition associated with abnormal aggregations of α-synuclein [12]. Five percent of non-institutionalized adults 85 years and older are believed to suffer from DLB, and the disease accounts for approximately 22% of all patients with dementia [35,36]. The fundamental features of DLB are dementia, fluctuating cognition (pseudodelerium), and visual hallucinations with Parkinsonism [36,37]. Frequently, patients with DLB have a rapid eye movement (REM) sleep behaviour disorder in the form of lively and often anxiety-filled dreams during the REM sleep phase, which may be accompanied by motor symptoms. This sleep behaviour disorder is characteristic for neurodegenerative disorders with pathological cerebral aggregates of α-synuclein [38,39].

**Figure 1:**
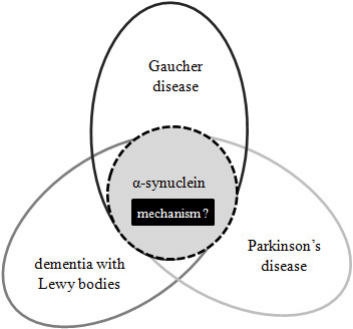
**Synucleinopathies demonstrating a relationship with Gaucher disease**.

Once the potential relationship between Parkinson's disease and Gaucher disease was evident, researchers expanded their investigations to assess whether *GBA *mutations were associated with other Lewy body disorders, such as DLB [40]. Initial findings from Goker-Alpan et al. [41] found *GBA *mutations in 23% of brain samples of 35 autopsy cases with DLB. A later study screening for only c.1448T > C (L444P) and c.1226A > G (N370S) mutations detected *GBA *alterations in 2 (3.5%) of 57 patients with DLB compared to 2 of 554 control subjects (0.4%) [29]. Subsequent studies reported mutations in *GBA *at frequencies ranging from 6% (n = 50) [42] to 28% (n = 95) [43] of DLB cases. Collectively, these genetic studies suggest that *GBA *mutations represent genetic risk factors for DLB [29,43]. Complementing these genetic investigations, Parnetti et al. [44] recently reported a pronounced decrease in GBA activity in cerebrospinal fluid of DLB patients. A similar reduction in GBA activity has been previously reported in PD [45]. This corroborates a relationship between Gaucher disease and the two aforementioned synucleinopathies, PD and DLB (Figure 1).

### Gaucher Disease, Multiple System Atrophy, and Neurodegeneration with Brain Iron Accumulation

Multiple system atrophy (MSA), a progressive neurodegenerative disorder, is characterized by autonomic failure, poor levodopa-responsive parkinsonism, cerebellar ataxia, and various pyramidal symptoms [46]. MSA-Parkinsonism type is the most common Western Hemisphere phenotype, while MSA-cerebellar type is predominant in the Eastern Hemisphere [47]. Mean survival is approximately nine to ten years after onset of symptoms [48], with nocturnal sudden death being a major cause of mortality [49,50]. MSA is commonly regarded as a primary oligodendrogliopathy due to widespread glial cytoplasmic inclusions [46,51]. These inclusions have demonstrated immunoreactivity for α-synuclein, thus relating MSA to other synucleinopathies such as Parkinson's disease and dementia with Lewy bodies [52-54]. Genetic studies have revealed that variants in the α-synuclein-encoding *SNCA *gene are major risk factors for MSA. Aside from the role of the *SNCA *gene, however, the etiopathogenesis of MSA has yet to be elucidated: interactions of genetic and environmental factors similar to other complex neurodegenerative diseases are probable [46,55].

Like MSA, neurodegeneration with brain iron accumulation (NBIA) falls under the synucleinopathy umbrella due to various reports of associated α-synuclein accumulation [56,57]. NBIA comprises a spectrum of progressive extrapyramidal disorders including the previously labelled Hallervorden-Spatz syndrome as well as additional disorders characterized by high levels of iron accumulation in the brain [58,59]. Determining whether a patient has NBIA and diagnosing a specific subtype may take several years, while the phenotype and radiographic changes evolve. The major form of NBIA, accounting for approximately 50% of cases, is pantothenate kinase-associated neurodegeneration (PKAN) caused by mutations in the *PANK2 *gene [58-60]. Other NBIA disorders, such as aceruloplasminaemia, which is caused by mutations in the *CP *gene, and neuroferritinopathy, which is caused by mutations in the *FTL *gene, appear to affect specific, small NBIA sub-populations [61,62]. The drive to identify major causative genes has helped refine the NBIA subtypes, providing clinicians with a systematic approach to diagnosing and treating these complex cases [59].

Though both NBIA and MSA are synucleinopathies, they do not exhibit the strong relationship with Gaucher disease seen in patients with Parkinson's disease or dementia with Lewy bodies. For NBIA, significant progress was made from 2009 to 2010 in differentiating subtypes according to genetic, radiologic, and clinical findings [63]. However, no correlation with GBA has been mentioned in the resulting literature. Interestingly, most Gaucher patients are anemic due to the presence of splenomegaly. Thus, they may have iron deficiency which could minimize their risk for NBIA. For MSA, numerous analyses have found that *GBA *mutations are not linked to the disease, suggesting that this branch of the ceramide pathway is unlikely to be associated with all types of primary α-synuclein deposition [41,46,64,65]. Therefore, for NBIA and MSA patients, there does not appear to be a need for modifying current genetic counselling approaches or for clinicians to perform additional inquiries about possible family members with Gaucher disease.

### Mechanism of Interaction

Exposure of the relationship between Gaucher disease, Parkinson's disease and dementia with Lewy bodies has generated a new challenge: to determine the mechanisms contributing to this association and why such an association does not extend to all synucleinopathies. Both gain-of- and loss-of-function explanations have been proposed [22]. Recently, a prion theory has also been suggested [66].

The gain-of-function theories have in common misfolded mutant glucocerebrosidase as the main culprit. Misfolded GBA has been suggested to contribute to neurodegeneration by inducing lysosomal insufficiency, by impairing autophagic pathways necessary for degrading α-synuclein, or by overburdening the ubiquitin-proteasome pathway [22,67]. Using cellular and *in vivo *models, Cullen et al. [68] recently analyzed the effects of wild-type and mutant GBA on α-synuclein. Results indicated that GBA mutants promoted α-synuclein accumulation in a dose- and time-dependent manner. In cell culture models, the gain-of-function toxic effect was mitigated by rapamycin.

According to the loss-of-function hypothesis, *GBA *haploinsuffiency might cause its substrate glucocerebroside and other polyunsaturated lipids to accumulate, altering the cell membrane sphingolipid composition. Subsequently, this could disrupt membrane binding of α-synuclein, increasing its aggregation in the cytoplasm [22,69,70]. Alternatively, elevated levels of glucocerebrosides could cause ryanodine receptor activation, leading to a rise in intracellular free calcium, followed by cell death and parkinsonism [66,71]. Mazzulli et al. [72] recently proposed a more comprehensive mechanism whereby deficient GBA leads to the accumulation of glucocerebroside in neurons that in turn promotes the formation of toxic α-synuclein oligomers. Elevated levels of the toxic α-synuclein species trigger depletion of lysosomal GBA and further stabilization of the α-synuclein oligomers by glucocerebroside accumulation, resulting in a self-propagating positive feedback loop leading to neurodegeneration.

Another theory gaining momentum is the possibility that PD is a prion disorder resulting from amplified production and/or impaired clearance of α-synuclein, prompting misfolding and the development of toxic oligomers, aggregates, and cell death. Moreover, it is feasible that α-synuclein itself is a prion protein that can self-aggregate and be transmitted to unaffected cells, thus propagating the disease process [73]. The Gaucher cell environment created by mutated glucocerebrosidase could serve as a vehicle to enhance these events [66].

The aforementioned models, however, all exhibit limitations. None can singlehandedly explain why only a fraction of those with *GBA *mutations actually develop PD or why carriers or patients with null *GBA *alleles can develop parkinsonian phenotypes. Westbroek et al. [74] suggest that the presence of aberrant glucocerebrosidase and/or subsequent changes in enzyme activity and substrate accumulation add to the pathology of α-synuclein in a secondary fashion. Hence, *GBA *mutations may augment rather than initiate α-synuclein pathology. Conversely, Sardi et al. [75] provide *in vivo *evidence that a single point mutation in *GBA *can cause α-synuclein misprocessing and cognitive deficits characteristic of synucleinopathies. Both enzymatic loss-of-function and toxic gain-of-function mechanisms were found to contribute to the development of the Gaucher-related synucleinopathies, and exogenous administration of glucocerebrosidase corrected the observed pathological features. Interestingly, Choi et al. [76] recently reported that patients with GBA-associated synucleinopathies showed aggregation of oligomeric forms of α-synuclein in SDS-soluble brain fractions, while only monomeric forms of α-synuclein were present in subjects with *GBA *mutations without parkinsonism.

## Conclusions

The high frequency of glucocerebrosidase mutations among ethnically diverse cohorts of Parkinson's disease patients render mutations in this gene as one of the most common and universally reported risk factors for PD [22]. It is also clear that a relationship exists between Gaucher disease and dementia with Lewy bodies. However, this association does not appear to extend to all synucleinopathies. Presently, no link has been found between *GBA *mutations and multiple system atrophy or neurodegeneration with brain iron accumulation.

The clinical implications of this relationship, such as modifications to genetic counseling or testing regimens, will need to be addressed. Hruska et al. [77] recommended that questions regarding parkinsonian symptoms be included in Gaucher patient evaluations and that inquiries about relatives with Gaucher disease be made in Parkinson disease clinics. However, caution was advocated in translating the findings to the patient community due to the low combined incidence and the potential to generate alarm.

The mechanism behind the relationship between *GBA *mutations and PD or DLB remains elusive. Gain-of-function, loss-of-function, and prion theories have been proposed. A better understanding of this link will provide new avenues for investigation, further clarification of synucleinopathy family members, and the development of novel therapies.

## List of Abbreviations

FDA: (US Food and Drug Administration); GBA: (glucocerebrosidase); NAC: (nonamyloid component); PD: (Parkinson's disease); DLB: (dementia with Lewy bodies); REM: (rapid eye movement); MSA: (multiple system atrophy); NBIA: (neurodegeneration with brain iron accumulation).

## Competing interests

A manuscript editing fee was provided to TNC from FYMC's Natural Sciences and Engineering Research Council grant #138216-2009. No conflict of interest is present.

## Authors' contributions

TNC was involved in the conception, researching and writing of the manuscript. FYMC provided expert content review. Both authors read and approved the final manuscript.
